# Commentary: Intercultural Competence Past, Present, and Future: Respecting the Past, Problems in the Present, and Forging the Future

**DOI:** 10.3389/fpsyg.2022.836372

**Published:** 2022-04-04

**Authors:** Mengmeng Bo, Gift Chinemerem Onwubuya

**Affiliations:** Faculty of Education, Shanghai Normal University, Shanghai, China

**Keywords:** intercultural competence, second language education, teacher education, intercultural communication, culture and language pedagogy

Since the 1980s, intercultural competence (IC) has been widely regarded as the main objective of teaching and learning foreign languages (Byram, 1997). Even though previous research shows that “teaching for linguistic competence cannot be separated from teaching for intercultural competence” (Byram, 1997), quite a few teachers still need professional support and guidance when it comes to the “how.” *Intercultural Competence Past, Present and Future: Respecting the Past, Problems in the Present and Forging the Future*, edited by María Dolores López-Jiménez and Jorge Sánchez-Torres helps L2 teachers understand how to integrate IC into language teaching. Apart from an introductory chapter, the remaining 11 chapters of this book can be divided into three thematic parts, which deal with IC issues in the past, present, and future. In this book, IC is “an umbrella term not only for “intercultural communicative competence” (ICC), “intercultural interaction competence” (ITC), and “intercultural communication” but also for any issue dealing with aspects that involve more than one specific culture” (p. 5).

Chapters 2 and 3 focus on heritage language (HL) learners and their learning motivation in the light of IC in the past. Chapter 2 intends to understand what motivates young learners to attend community heritage language (CHL) schools and how these learners in CHL schools develop their intercultural awareness. In the findings, HL learners are largely motivated by the desire to communicate with their community members and the potential use of the language in professional and career domains. The ethnographic and longitudinal case study in Chapter 3 aims to address whether year-long sojourners' experiences could improve their language and cultural proficiency in the host country, which buttresses the individual, intricate, and multidimensional mechanism of adapting to the language and cultural dimension of life in the host country as an HL learner. The study results add more questions to the broad view that immersion always occurs when L2 learners are physically located in the host community.

Chapters 4–7 address a different theme through a collection of English language teachers' research in different cultural contexts, searching for IC pedagogical implications in L2 teacher practice. Chapter 4 reveals the pedagogical problems of the misleadingly stereotyped images of international students (for example, Asian students) enrolled in an Australian university. It advocates a negotiated language that benefits mutual understanding, and reports positive changes in international students' interactions through critical intercultural awareness and communication in English classes. Chapter 5 is a comparative study exploring the relationship between teacher confirmation and student motivation in the US and Finland; we find that American students are more likely to be motivated by perceived teacher confirmation.

The next chapter revolves around how students with different cultural backgrounds construct feedback on their oral expressions. The findings demonstrate that teachers play an extremely weak role in helping international students understand feedback. In addition, teachers' intercultural sensitives grow out of their construction but not intercultural negotiation. Chapter 7 sheds light on teachers' reflection upon intercultural language education, indicating that teachers' reflection assists international students in knowing more about the host culture than the target culture. Unfortunately, teachers do not apply reflection to target language teaching practice.

The last part of the book links to the possible pedagogical models in near future. The first two chapters of this part focus on L2 teachers' perceptions of IC and critical cultural awareness. Chapter 10 presents how online courses enhance interculturality and avoid stereotyping. Chapter 11 and 12 discuss the positive effects of two instructional tools for teaching/learning a C2: an Autobiography of Intercultural Encounters through visual media (AIEVM) and a decoding of pork, alcohol, religion, sex, narcotics, ism, and politics (PARSNIP). AIEVM is found to be effective in encouraging students to reflect on otherness by image. In contrast, PARSNIP is helpful in promoting students' IC, and enhancing English teachers' awareness of their role in the age of globalization.

In summary, by unveiling the underlying problems present in L2 teaching and learning at various school levels in different nations, this book makes a timely contribution to the study of IC in language education. It presents global English teachers with specific implementation tools in their real teaching practice. Principals' leadership may also benefit from this volume as it can inspire them to shape post-service training of IC catering to English teachers' real needs. Besides, the theoretical implications of this book might be of high interest for researchers to examine teacher education from a post-structuralist theoretical perspective, which emphasizes reflection and human experience (p. 277), insists on listening to the voices of “realist tales” (Maanen, 2011) and rejects the “hegemonic grip of the hypothetico-deductive method of scientific investigation” (Guba and Lincoln, 1994).

However, readers might find this book more helpful with all the merits mentioned above if it had encompassed more empirical studies on Asia and Africa. Furthermore, methods of motivating L2 teachers' self-research consciousness in effectively combining IC with L2 teaching should be explored in future research, as pedagogical evolution starts with teachers' agency. Moreover, some chapters add nuances to language learning motivation and L2 acquisition studies. Since learners' motivation is crucial in any learning setting, new paradigms on the relationship between learners' demotivation and IC also need to be explored in the near future. Last but not the least, going beyond the framework of linguistics, future IC research is becoming interdisciplinary and calls for attention from psychology, sociology, and other disciplines (p. 279).

## Author Contributions

MB conceived of the presented idea and outlined the structure of the book review and has done every part of the manuscript. GO helps polishing the writing language. All authors discussed the comments and contributed to the final manuscript. All authors contributed to the article and approved the submitted version.

## Conflict of Interest

The authors declare that the research was conducted in the absence of any commercial or financial relationships that could be construed as a potential conflict of interest.

## Publisher's Note

All claims expressed in this article are solely those of the authors and do not necessarily represent those of their affiliated organizations, or those of the publisher, the editors and the reviewers. Any product that may be evaluated in this article, or claim that may be made by its manufacturer, is not guaranteed or endorsed by the publisher.
